# Antibody–Drug Conjugates Targeting Resistance-Associated Signaling Pathways: Recent Advances and Future Perspectives

**DOI:** 10.3390/ijms27073287

**Published:** 2026-04-04

**Authors:** Dan Xie, Chengming Yang, Siyi Gao, Jinqian Li, Jiaping Yang, Xinhao Li, Ruoyu Jiang, Fangyu Cao, Sheng Zhang, Lianghua Wang, Mingjuan Sun

**Affiliations:** 1 Department of Biochemistry and Molecular Biology, Naval Medical University, Shanghai 200433, China; danxie@smmu.edu.cn (D.X.); nicecanny@sina.cn (S.G.); 18766738223@163.com (J.L.); smmu_shenghuayjp@163.com (J.Y.); hydlxh@smmu.edu.cn (X.L.); jiangruoyu0621@163.com (R.J.); cfytgzy@163.com (F.C.); 2 Department of Student Team, College of Basic Medical Sciences, Naval Medical University, Shanghai 200433, China; cmyang0903@163.com; 3 Medical Oncology, Shanghai Cancer Center, Fudan University, Shanghai 200032, China; zhang_s@fudan.edu.cn

**Keywords:** ADCs, drug resistance, signaling pathways, malignant tumors

## Abstract

Antibody–drug conjugates (ADCs) represent a paradigm shift in precision oncology, ingeniously coupling the targeting capability of monoclonal antibodies with the lethal potency of cytotoxic payloads to selectively eradicate tumor cells. While ADCs have demonstrated transformative efficacy across a spectrum of malignancies, the emergence of intrinsic and acquired resistance remains a formidable obstacle, frequently culminating in treatment failure and disease progression. The landscape of ADC resistance is highly complex, governed by a diverse array of molecular mechanisms. These range from alterations in antigen dynamics—such as downregulation or impaired trafficking—to intracellular adaptations, including the upregulation of multi-drug resistance efflux pumps, enhanced DNA damage repair capacity, and the blockade of apoptotic cell death. Moreover, tumor cells often exploit compensatory signaling networks to bypass therapeutic inhibition. Consequently, elucidating the intricate signaling cascades that drive these resistance phenotypes is critical for clinical advancement. This review comprehensively examines the pivotal signaling pathways underpinning ADC resistance and evaluates novel therapeutic strategies designed to circumvent these molecular barriers, aiming to optimize patient outcomes.

## 1. Introduction

**Figure 1:**
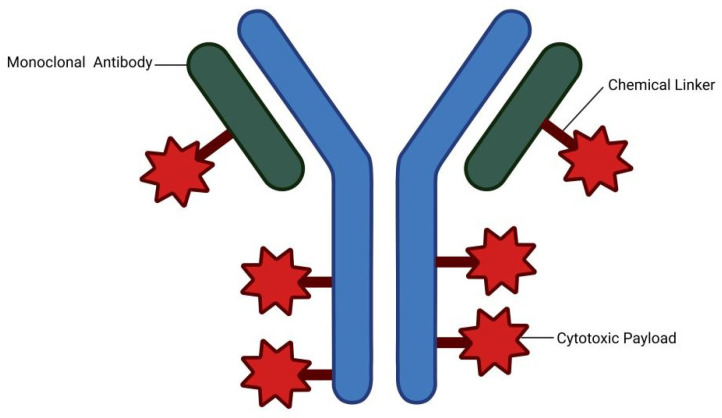
Antibody–drug conjugate structure.

ADCs deliver cytotoxic payloads by specifically targeting tumor-associated antigens. This strategy enhances the therapeutic index and can mediate a potent bystander effect, eliminating adjacent antigen-negative cancer cells [1,2,3,4]. As shown in Figure 1, in contrast to conventional chemotherapy, ADCs leverage the targeting specificity of monoclonal antibodies to selectively deliver cytotoxic agents to tumor tissues. This dual mechanism of action significantly enhances antitumor efficacy while minimizing off-target toxicity, establishing ADCs as a pivotal approach in the field of precision oncology [5]. The clinical versatility of ADCs is evident in their success across diverse malignancies. Since the initial Food and Drug Administration (FDA) approval in 2000 of the first ADC, gemtuzumab ozogamicin (GO), for the treatment of CD33-positive acute myeloid leukemia (AML) [6], a substantial number of ADCs have subsequently been approved to treat both hematological malignancies and solid tumors. For instance, brentuximab vedotin (BV) has redefined the therapeutic standard for relapsed or refractory Hodgkin lymphoma (HL) and systemic anaplastic large cell lymphoma (sALCL) [7]. Similarly in solid tumors, trastuzumab emtansine (T-DM1) has established itself as a cornerstone therapy for Human epidermal growth factor receptor 2 (HER2)-overexpressing breast cancer (BC) [8,9]. Currently, the ADC field continues to advance rapidly, with a growing pipeline of novel candidates under clinical investigation.

Despite the notable progress achieved with ADCs, their therapeutic efficacy remains limited by several factors, including dose-limiting toxicity, linker instability, and antibody immunogenicity. Among these limitations, acquired resistance stands as a paramount challenge, which can undermine the durability of treatment responses through diverse mechanisms such as altered antigen expression, upregulation of drug efflux pumps, and tumor heterogeneity [10]. To address these limitations, recent research efforts are focused on developing more precise strategies, including next-generation targeting technologies, antibody engineering, tumor microenvironment (TME) modulation, and rational combination therapies. However, to successfully advance the design and clinical translation of ADCs, it is essential to first delineate the intracellular cascades that tumor cells exploit to counteract ADC efficacy. Therefore, in the following sections, we will explore the key signaling pathways associated with ADC resistance—namely, the PI3K/AKT/mTOR, MAPK/ERK, Wnt/β-catenin, RTK, and JAK/STAT3 pathways—discussing how their dysregulation contributes to treatment failure and how they might be targeted to enhance ADC performance.

This review systematically summarizes the latest progress in understanding ADC resistance mechanisms. Aberrant activation of the aforementioned signaling pathways constitutes a core molecular basis for resistance to ADC therapy. First, as a critical regulator of cell proliferation, survival, and metabolism, hyperactivation of the PI3K/AKT/mTOR pathway mediates ADC resistance by upregulating drug efflux pumps (e.g., P-glycoprotein [P-gp]), promoting epithelial–mesenchymal transition (EMT), and inhibiting apoptosis. This mechanism is particularly relevant in breast cancer—a primary indication for HER2-targeted ADCs—where mutations in this pathway are observed in approximately 25% to 40% of cases, frequently driving acquired resistance [11]. Second, compensatory activation of the MAPK/ERK signaling pathway represents another critical resistance mechanism across various tumor types. When primary signaling pathways are inhibited by an ADC, tumor cells often sustain survival signals by activating the RAS-RAF-MEK-ERK cascade. This pathway plasticity significantly contributes to the limited durability of responses observed in single-target ADC therapies [12,13]. Third, persistent hyperactivation of the Wnt/β-catenin signaling pathway plays a pivotal role in tumorigenesis, therapeutic resistance, and the maintenance of cancer stem cell properties. In solid tumors frequently targeted by emerging ADCs, such as non-small cell lung cancer (NSCLC), this pathway actively contributes to treatment failure by preserving cancer stemness and promoting EMT [14,15]. Fourth, the redundancy and compensatory activation of receptor tyrosine kinase (RTK) networks constitute a broad mechanism of ADC resistance. The epidermal growth factor receptor (EGFR) family members—including EGFR, HER2, HER3, and HER4—represent a critical subclass of RTKs. Across many epithelial tumors, their aberrant activation and crosstalk with downstream effectors, such as the PI3K/AKT and MAPK pathways, create a complex network that bypasses ADC-induced cytotoxicity [16,17]. Finally, activation or dysregulation of the JAK/STAT3 signaling pathway mediates resistance by promoting cell survival, proliferation, and immune evasion. In malignancies such as bladder cancer—where ADCs have recently become standard-of-care therapies—aberrant JAK/STAT3 signaling, combined with its crosstalk with the PI3K/AKT and MAPK pathways, further compounds the complexity of the resistance landscape [18,19]. Ultimately, the extensive crosstalk and compensatory mechanisms among these signaling pathways form a complex resistance network. Understanding this network provides a critical theoretical foundation and informs target selection for developing next-generation ADC technologies and rational combination therapies.

## 2. ADC and Its Drug Resistance Classification

### 2.1. Fundamentals of ADCs: Structure and Mechanism of Action

Antibody–drug conjugates (ADCs) represent an important class of targeted anticancer therapeutics designed to combine the tumor specificity of monoclonal antibodies (mAb) with the potent cytotoxic activity of small-molecule drugs. Structurally, an ADC is composed of three essential components: a mAb, a chemical linker, and a cytotoxic payload. The antibody component selectively recognizes tumor-associated antigens that are preferentially expressed on the surface of malignant cells, thereby enabling targeted delivery. The linker covalently connects the antibody to the cytotoxic payload and plays a critical role in determining both the stability of the ADC in circulation and the controlled release of the drug within tumor cells. The payload generally consists of highly potent cytotoxic agents, such as microtubule inhibitors or DNA-damaging compounds, which are capable of inducing cell death at extremely low concentrations [20,21] (Figure 1).

The therapeutic activity of ADCs relies on a multistep biological process. First, the antibody moiety binds to its corresponding antigen expressed on the tumor cell surface. The resulting antigen–ADC complex is subsequently internalized through receptor-mediated endocytosis and trafficked to intracellular compartments such as endosomes and lysosomes. Within these compartments, the linker undergoes enzymatic or chemical cleavage, leading to the release of the cytotoxic payload into the cytoplasm. Once released, the payload disrupts essential cellular processes, including microtubule polymerization or DNA replication, thereby inducing apoptosis or other forms of programmed cell death. In addition, certain payloads possess membrane permeability, allowing them to diffuse into neighboring tumor cells and produce a bystander effect, which may further enhance therapeutic efficacy in tumors with heterogeneous antigen expression [20,21].

Despite significant improvements in the design of ADCs in recent years, the therapeutic outcome of ADC-based therapies depends on the coordinated performance of multiple biological and pharmacological steps. Disruptions occurring at any stage of the ADC delivery process—from antigen recognition to payload activity—may compromise treatment efficacy and ultimately contribute to therapeutic resistance [22].

### 2.2. Classification of ADC Resistance: Primary and Secondary Resistance

Despite the substantial clinical success of several ADCs across multiple malignancies, therapeutic resistance has emerged as a major challenge limiting their long-term efficacy. Similar to other targeted therapies and cytotoxic agents, resistance to ADCs can generally be categorized into two major types: primary (intrinsic) resistance and secondary (acquired) resistance [23]. Primary resistance refers to the intrinsic insensitivity of tumor cells to ADC therapy prior to treatment initiation. This form of resistance is often associated with unfavorable tumor characteristics that prevent effective ADC action. For example, insufficient or heterogeneous expression of the target antigen may reduce antibody binding and limit ADC internalization. In addition, certain tumor cells may exhibit intrinsic resistance to the cytotoxic payload, particularly when mechanisms such as drug efflux or altered microtubule dynamics reduce payload activity [20]. In contrast, secondary resistance develops after an initial period of clinical response and arises during continued exposure to ADC therapy. Acquired resistance may result from adaptive changes in tumor cells that occur under therapeutic pressure. Several mechanisms have been proposed, including the downregulation or loss of target antigen expression, alterations in intracellular trafficking pathways, impaired lysosomal processing, and activation of compensatory survival signaling pathways. Furthermore, enhanced drug efflux mediated by ATP-binding cassette (ABC) transporters may reduce the intracellular accumulation of released payloads, thereby attenuating cytotoxic activity [22]. Next, we will discuss resistance mechanisms associated with the activation of adaptive signaling pathways.

### 2.3. Tumor-Intrinsic Mechanisms of ADC Resistance

Accumulating evidence suggests that multiple tumor-intrinsic factors can interfere with the pharmacological activity of ADCs. These mechanisms may operate at different stages of the ADC delivery and action process, including antigen recognition, intracellular trafficking, payload release, and cytotoxic activity [22]. One important resistance mechanism involves alterations in target antigen expression. Effective ADC therapy relies on sufficient antigen density on the tumor cell surface to enable efficient antibody binding and internalization. However, tumor cells may exhibit heterogeneous antigen expression or undergo adaptive downregulation of the target antigen during treatment. Such changes reduce ADC binding and limit intracellular drug delivery, thereby diminishing therapeutic efficacy [20,22,24,25]. Another mechanism involves defects in ADC internalization and intracellular trafficking. Following antigen binding, ADCs must be internalized through receptor-mediated endocytosis and transported to lysosomal compartments for payload release. Disruptions in endocytic pathways or intracellular trafficking processes may impair this transport, preventing ADCs from reaching lysosomes and consequently reducing payload liberation [26]. Lysosomal dysfunction also represents a potential barrier to effective ADC activity. The release of cytotoxic payloads often depends on proteolytic cleavage or chemical degradation within the lysosomal environment. Alterations in lysosomal pH, reduced protease activity, or other changes in lysosomal function may compromise linker cleavage and payload release [27,28]. Finally, resistance may arise through increased drug efflux and cellular detoxification mechanisms. Many cytotoxic payloads used in ADCs are substrates for ATP-binding cassette transporters such as P-glycoprotein (MDR1). Overexpression of these transporters can promote the active efflux of cytotoxic drugs from tumor cells, thereby reducing intracellular drug concentrations and limiting antitumor activity [29,30,31].

Based on those, this review next discusses the relationship between ADC resistance-specific processes such as payload sensitivity, efflux, and anti-apoptotic protection and the activation of signaling pathways.

## 3. Signaling Pathways in ADC Resistance

### 3.1. The PI3K/AKT/mTOR Signaling Pathway

#### 3.1.1. Structural Basis and Biological Functions of the PI3K/AKT/mTOR Signaling Pathway

The phosphatidylinositol-4,5-bisphosphate 3-kinase/protein kinase B/mammalian target of rapamycin (PI3K/AKT/mTOR) signaling axis acts as a foundational intracellular survival network. In the context of ADCs, its hyperactivation constitutes one of the most formidable biological barriers to therapeutic efficacy. This structural cascade is typically initiated by surface receptor tyrosine kinases (RTKs), such as HER2. Upon activation, PI3K converts phosphatidylinositol 4,5-bisphosphate (PIP2) to phosphatidylinositol (3,4,5)-trisphosphate (PIP3), which subsequently recruits AKT to the plasma membrane. AKT achieves full oncogenic activation through phosphorylation by phosphoinositide-dependent kinase-1 (PDK1) and the mTOR complex 2 (mTORC2) [32,33,34].

Rather than viewing PI3K and AKT as isolated oncogenic drivers, it is critical to understand them as a highly coordinated anti-apoptotic engine. When this pathway undergoes aberrant activation—frequently driven by underlying PIK3CA mutations or the loss of the tumor suppressor phosphatase and tensin homolog (PTEN) [35,36,37,38]—it fundamentally rewires the tumor cell’s survival circuitry. Activated AKT actively suppresses pro-apoptotic signals while simultaneously hyper-stimulating mTOR complex 1 (mTORC1), which drives aggressive protein synthesis and metabolic reprogramming via ribosomal protein S6 kinase beta-1 (S6K1) and eukaryotic translation initiation factor 4E-binding protein 1 (4EBP1). For ADC therapies, this establishes a robust bypass mechanism. Consequently, even when the antibody component successfully engages its target and releases its cytotoxic payload (e.g., DM1) into the cytoplasm, the pre-activated survival signaling directly neutralizes the payload-induced apoptotic triggers [39,40,41,42,43].

The PI3K signaling cascade is primarily initiated by the activation of receptor tyrosine kinases such as HER2, which recruits PI3K to convert PIP2 into PIP3. This reaction is negatively regulated by the tumor suppressor PTEN. The accumulation of PIP3 recruits Akt and PDK1 to the plasma membrane, where Akt is phosphorylated and activated by PDK1 and the mTORC2 complex. Activated Akt subsequently stimulates mTORC1, which regulates downstream effectors like S6K1 and 4EBP1 to drive cell proliferation, metabolism, and survival. However, in the context of targeted therapy, the ADC T-DM1 binds to HER2 and undergoes internalization and lysosomal degradation. This process releases a cytotoxic payload that disrupts microtubules, leading to cell death. Nevertheless, upon lysosomal degradation of T-DM1, the cytotoxic payload is released. However, resistance is mediated by the upregulation of ABC transporters (Efflux Pumps) on the lysosomal or plasma membrane. Instead of allowing the payload to bind microtubules (indicated by the X and blocked pathway), these pumps actively divert and efflux the drug away from its target, reducing the intracellular concentration required to induce apoptosis. Resistance can occur through mechanisms such as drug efflux pumps or constitutive PI3K pathway activation. To counteract this, the specific inhibitor Alpelisib targets PI3K, blocking the signaling cascade and overcoming resistance. Through these combined mechanisms, the modulation of HER2 and PI3K signaling plays an essential role in determining the balance between tumor cell survival and therapeutic-induced apoptosis. HER2: human epidermal growth factor receptor 2; T-DM1: trastuzumab emtansine; PI3K: phosphoinositide 3-kinase; PIP2/3: phosphatidylinositol (4,5)-bisphosphate/(3,4,5)-trisphosphate; PTEN: phosphatase and tensin homolog; Akt: protein kinase B; mTORC1/2: mammalian target of rapamycin complex 1/2; PDK1: phosphoinositide-dependent kinase-1.

#### 3.1.2. Critical Role of the PI3K/AKT/mTOR Signaling Pathway in Treatment Resistance of HER2-Positive Breast Cancer

The PI3K/AKT/mTOR Signaling Pathway hyperactivation promotes survival, reduces apoptosis triggered by payloads, and upregulates ABC transporters, thereby limiting payload accumulation. In HER2-targeted ADCs, PIK3CA mutations or PTEN loss attenuate response by sustaining survival despite payload delivery [44]. In HER2-positive breast cancer, the PI3K/AKT/mTOR pathway represents a primary bottleneck to ADC efficacy, with approximately 30–40% of cases exhibiting constitutive activation of this network [45,46]. This activation severely limits the durability of ADCs like T-DM1. Because PIK3CA mutations and PTEN loss provide a parallel survival route that completely bypasses the HER2 blockade, combined molecular profiling is increasingly utilized to predict early disease progression [47].

**Figure 2:**
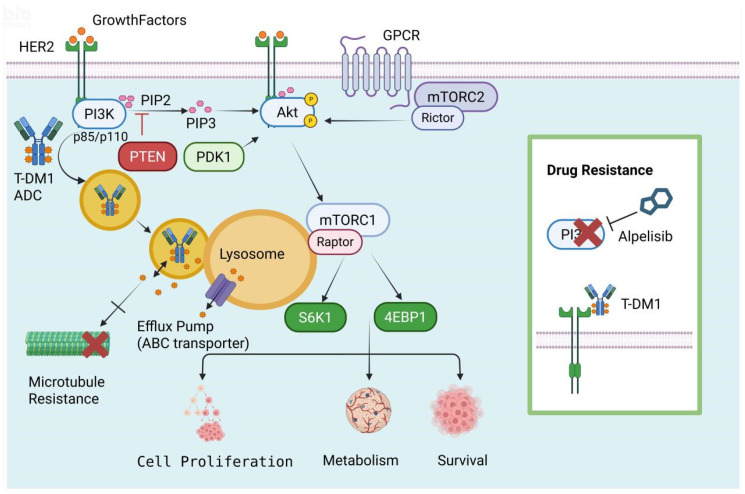
The HER2-PI3K-Akt-mTOR signaling axis and T-DM1 mechanism of action.

The clinical necessity of targeting this bypass track is evidenced by trials combining the PI3Kα inhibitor alpelisib with T-DM1 in refractory HER2-positive metastatic breast cancer. This combination yielded an objective response rate (ORR) of 43% and a clinical benefit rate (CBR) of 71%. Crucially, even in a subgroup of patients who had already progressed on prior T-DM1 monotherapy, the combination achieved a 30% ORR and 60% CBR, alongside a median progression-free survival (PFS) of 8.1 months [47]. Analytically, these findings do more than demonstrate safety; they provide critical proof-of-concept that pharmacological blockade of the PI3K axis dismantles the compensatory survival network, forcibly resensitizing highly refractory tumors to ADC-mediated cytotoxicity (Figure 2).

#### 3.1.3. Research Status of PI3K/AKT/mTOR Signaling Pathway-Driven ADC Resistance

The PI3K/AKT/mTOR signaling pathway serves as a critical driver of ADC resistance. While ADCs are designed to deliver cytotoxic payloads that trigger apoptosis, aberrant activation of the PI3K pathway provides robust counter-regulatory survival signals [48,49]. While ADCs rely on delivering potent cytotoxic payloads to trigger irreversible DNA damage or microtubule disruption, aberrant AKT/mTOR signaling actively counteracts these effects at multiple cellular levels.

Specifically, hyperactive AKT upregulates anti-apoptotic proteins like B-cell lymphoma 2 (Bcl-2) and promotes the transcription of ATP-binding cassette (ABC) transporters, such as P-glycoprotein (P-gp) [50]. This creates a highly efficient drug-efflux environment. Mechanistically, this means that even if the ADC is successfully internalized and the linker cleaved, the payload is rapidly pumped out of the cytoplasm before reaching a lethal threshold. Furthermore, PI3K hyperactivation induces EMT, shifting cells into a dormant, drug-tolerant state. Consequently, deploying specific PI3K/AKT/mTOR inhibitors alongside ADCs is not merely an additive therapy, but a necessary intervention to suppress efflux pump expression, restore apoptotic sensitivity, and maximize payload retention [51] (Table 1).

### 3.2. The MAPK/ERK Signaling Pathway

#### 3.2.1. Structural Features and Oncogenic Mechanisms of the MAPK/ERK Signaling Pathway

The mitogen-activated protein kinase/extracellular signal-regulated kinase (MAPK/ERK) signaling pathway, operating primarily through the classic rat sarcoma virus (RAS) to rapidly accelerated fibrosarcoma (RAF) to MAPK/ERK kinase (MEK) enzymatic cascade, is a critical regulator of cellular proliferation and a dominant mediator of ADC resistance [75,76,77,78]. Oncogenic hyperactivation of this pathway generally occurs via intrinsic genetic mutations (e.g., KRAS or BRAF) [79,80,81] or dynamic “bypass signaling” triggered by the compensatory upregulation of alternative RTKs—such as mesenchymal–epithelial transition factor (MET), EGFR, or fibroblast growth factor receptor (FGFR) [82,83,84].

**Figure 3:**
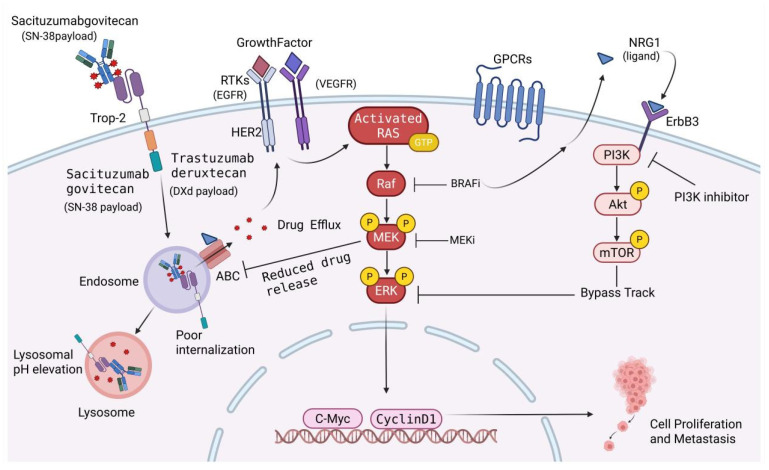
The MAPK/ERK Signaling Pathway and mechanisms of ADC resistance.

When exposed to the cytotoxic insult of an ADC, sustained ERK signaling provides an immediate evolutionary advantage. By driving the overexpression of pro-proliferative transcription factors while inhibiting apoptotic effectors, the pathway establishes a robust cellular defense. Furthermore, the MAPK pathway shares extensive crosstalk with the PI3K/AKT axis, creating redundant, failsafe signaling networks. This redundancy ensures that even if an ADC successfully engages its primary surface target, downstream proliferative signals remain uninterrupted, ultimately culminating in treatment failure [85,86]. (Figure 3).

Cellular signaling is primarily driven by surface receptors such as Trop-2, RTKs (including EGFR, HER2, and VEGFR), GPCRs, and ErbB3. Upon activation by ligands like Growth Factors or NRG1, Activated RAS triggers the Raf-MEK-ERK cascade, while parallel activation of the PI3K-Akt-mTOR pathway occurs. These signals converge in the nucleus to upregulate C-Myc and CyclinD1, promoting cell proliferation and metastasis. However, ADCs such as Sacituzumab govitecan (carrying SN-38) and Trastuzumab deruxtecan (carrying DXd) target surface antigens to deliver cytotoxic payloads. Following binding, the ADC undergoes internalization and trafficking to the lysosome for drug release. Nevertheless, resistance can occur through mechanisms such as poor internalization, lysosomal pH elevation, or drug efflux via ABC transporters. Additionally, therapeutic pressure from BRAFi, MEKi, or PI3K inhibitors can lead to the activation of bypass tracks, restoring signaling flux. Through these pathways, the interplay between drug delivery and signaling plasticity plays an essential role in tumor survival and treatment failure. Trop-2: trophoblast cell-surface antigen 2; RTKs: receptor tyrosine kinases; EGFR: epidermal growth factor receptor; VEGFR: vascular endothelial growth factor receptor; GPCRs: G-protein-coupled receptors; NRG1: neuregulin 1; RAS: rat sarcoma virus; MEK: mitogen-activated protein kinase kinase; ERK: extracellular signal-regulated kinase; ABC: ATP-binding cassette transporters; SN-38: active metabolite of irinotecan; DXd: deruxtecan.

#### 3.2.2. MEK-Mediated Feedback Activation and Drug Resistance Mechanisms

Accumulating evidence suggests that the MAPK/ERK pathway plays a pivotal role in acquired resistance to ADCs. On one hand, activation of this pathway upregulates drug efflux pumps such as ABC transporters, thereby reducing intracellular payload accumulation [87]. On the other hand, it triggers downstream survival signals that compensate for payload-induced mitotic catastrophe, protecting tumor cells from cytotoxic damage [88]. These dual mechanisms highlight the MAPK/ERK pathway as a potential therapeutic target to overcome ADC resistance. Moreover, a major challenge in overcoming MAPK-driven resistance is the pathway’s inherent capacity for rapid feedback activation. When primary signaling nodes like BRAF or MEK are pharmacologically inhibited, tumor cells often lack the physiological negative feedback loops necessary to maintain suppression, resulting in a swift, adaptive rebound [54].

This adaptive plasticity has profound implications for ADC therapies. For example, Fattore et al. demonstrated that inhibiting the BRAF/MEK cascade triggers a massive upregulation of neuregulin 1 (NRG1) secretion, leading to hyperphosphorylation of the erythroblastic oncogene B 3 (ErbB3/HER3) receptor. This acutely shifts the tumor’s survival dependency to the PI3K/AKT axis. Strikingly, combining kinase inhibitors with neutralizing antibodies against ErbB3 completely dismantled this compensatory loop, dramatically lowering the half-maximal inhibitory concentration (IC50) values of the therapies [55]. For ADC development, these findings emphasize that tumors respond to targeted inhibition by rapidly remodeling their surface receptor profiles. This bypass activation actively disrupts the internalization dynamics of ADCs and alters lysosomal pH, significantly blunting the payload’s impact [56].

#### 3.2.3. Strategies and Prospects for Overcoming Drug Resistance Through Combination Therapy

To circumvent the profound adaptability of the MAPK pathway, future ADC strategies must move beyond monotherapy and adopt rational combination frameworks. Preclinical evidence strongly supports the synergistic potential of combining ADCs—such as those targeting trophoblast cell-surface antigen 2 (Trop-2)—with MEK inhibitors to prevent the emergence of early survival feedback loops [56,57].

The necessity of multi-node blockade is underscored by findings that tumor cells can acquire concurrent mutations (e.g., MEK2 and BRAF aberrations), creating a hyper-resilient phenotype that requires simultaneous MAPK and PI3K/mTOR inhibition [89]. In the context of ADCs, sustained MAPK activation not only drives proliferation but actively alters the physical mechanisms of ADC efficacy by downregulating target antigen expression. Notably, metabolic adaptations, such as altered cholesterol metabolism, often fuel this feedback activation [58]. Ultimately, combining ADCs with MEK inhibitors or metabolic modulators represents a highly promising strategy to stabilize antigen expression, optimize lysosomal payload release, and dismantle compensatory survival networks (Table 1).

### 3.3. The JAK/STAT3 Signaling Pathway

#### 3.3.1. Molecular Structure and Activation Mechanism of the JAK/STAT3 Signaling Pathway

The Janus kinase/signal transducer and activator of transcription 3 (JAK/STAT3) signaling pathway serves as a master regulator of tumor inflammation, immune evasion, and cell survival, making its dysregulation a critical driver of ADC resistance [90,91,92]. Canonical activation is typically initiated by the binding of pro-inflammatory cytokines, most notably interleukin-6 (IL-6), to their surface receptors. This engagement activates JAKs, which subsequently phosphorylate STAT3, prompting its dimerization and translocation into the nucleus [93,94,95,96].

**Figure 4:**
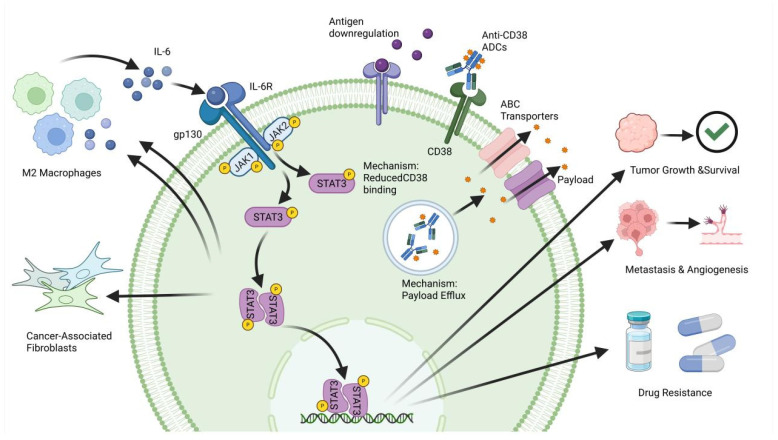
The IL-6/JAK/STAT3 signaling axis and anti-CD38 ADC mechanism of action.

Once in the nucleus, phosphorylated STAT3 orchestrates a massive transcriptional program that directly impedes ADC efficacy on two fronts. Intrinsically, it upregulates powerful anti-apoptotic proteins, such as B-cell lymphoma extra large (Bcl-xL) and myeloid cell leukemia 1 (Mcl-1), neutralizing the payload’s effects. Extrinsically, STAT3 reshapes the tumor microenvironment (TME) into an immunosuppressive fortress by recruiting myeloid-derived suppressor cells (MDSCs) and upregulating programmed death-ligand 1 (PD-L1). This effectively shuts down the localized “bystander effect” and secondary immune clearance that many ADCs rely upon for maximal tumor eradication [97,98,99,100] (Figure 4).

The JAK/STAT signaling cascade is primarily initiated by the release of IL-6 from tumor microenvironment components, such as M2 macrophages and cancer-associated fibroblasts. IL-6 binds to the IL-6 receptor (IL-6R) and gp130 complex, leading to the activation of receptor-associated kinases JAK1 and JAK2. These kinases phosphorylate STAT3, which subsequently dimerizes and translocates to the nucleus. In the nucleus, activated STAT3 regulates gene transcription to drive tumor growth, survival, metastasis, and angiogenesis. However, in the context of targeted therapy, anti-CD38 ADCs bind to surface CD38 and undergo internalization. This process releases a cytotoxic payload intended to disrupt cellular functions and induce cell death. Nevertheless, resistance can occur through mechanisms such as antigen downregulation (reducing CD38 binding sites) or the expulsion of the cytotoxic payload via ABC transporters. Through these combined mechanisms, the modulation of IL-6 signaling and transporter activity plays an essential role in determining therapeutic efficacy and the development of drug resistance. IL-6: interleukin-6; JAK: Janus kinase; STAT3: signal transducer and activator of transcription 3; ADC: antibody–drug conjugate; ABC: ATP-binding cassette; gp130: glycoprotein 130; IL-6R: interleukin-6 receptor.

#### 3.3.2. Mechanisms of ADC Resistance Mediated by the JAK/STAT3 Signaling Pathway

The JAK/STAT3 pathway mediates ADC resistance through a highly coordinated array of mechanisms spanning target modulation, TME remodeling, and enhanced drug efflux. First, the pathway directly modulates target availability. For instance, in multiple myeloma, hyperactive JAK/STAT3 signaling actively downregulates the surface expression of cluster of differentiation 38 (CD38). Because anti-CD38 ADCs depend on high antigen density for successful binding, this downregulation renders the tumor “invisible” to the drug. Conversely, pharmacological inhibition of STAT3 restores CD38 expression, directly rescuing ADC binding capacity [59].

Second, IL-6/STAT3 signaling facilitates continuous cross-talk between tumor cells and stromal components like cancer-associated fibroblasts (CAFs). This interaction creates a physical and biochemical niche that protects tumor cells from cytotoxic penetration [60,61,62,63,64]. Finally, STAT3 activation represents a primary transcriptional driver for ABC transporters, including ATP-binding cassette super-family G member 2 (ABCG2) and multidrug resistance-associated protein 1 (MRP1). By upregulating these highly efficient efflux pumps on the plasma and lysosomal membranes, the pathway ensures that internalized ADC payloads are aggressively expelled before inducing apoptosis [65].

Moreover activation of the JAK/STAT3 pathway has been implicated in acquired resistance to ADCs. Sustained STAT3 signaling upregulates anti-apoptotic genes of the Bcl-2 family, thereby blunting payload-induced apoptosis even after successful intracellular payload release [94].

#### 3.3.3. Therapeutic Strategies and Future Prospects for JAK/STAT3 Signaling Pathway-Mediated ADC Resistance

Understanding the multifaceted role of the JAK/STAT3 pathway in ADC resistance opens clear avenues for targeted interventions. Because this pathway drives resistance by stripping the tumor of target antigens, hyper-activating efflux pumps, and fostering an immunosuppressive stroma, it represents a high-value node for combination therapy. Co-administering JAK/STAT3 inhibitors with ADCs is emerging as a rational strategy to disrupt these tumor-supportive networks, “lock” target antigens on the cell surface, and break down microenvironment-mediated physical barriers, thereby profoundly amplifying the overall therapeutic index of the ADC [61,101,102,103] (Table 1).

### 3.4. The Wnt/β-Catenin Signaling Pathway

#### 3.4.1. Structural Basis and Biological Functions of the Wnt/β-Catenin Signaling Pathway

The Wingless-related integration site (Wnt)/β-catenin signaling pathway is a highly conserved network essential for tissue homeostasis; however, its aberrant reactivation in malignancies drives profound adaptive resistance and the maintenance of cancer stem cells (CSCs). The hallmark of this pathway’s activation is the inhibition of the destruction complex—comprising adenomatous polyposis coli (APC) and glycogen synthase kinase 3 beta GSK−3β—which prevents the ubiquitin-mediated degradation of β-catenin. Stabilized β-catenin translocates to the nucleus, complexing with T-cell factor/lymphoid enhancer factor (TCF/LEF) transcription factors to initiate oncogene expression [104,105,106,107].

This transcriptional reprogramming induces a dedifferentiated, stem-like state within the tumor. Because ADC payloads primarily target rapidly dividing, differentiated cells, the slow-cycling CSC populations fostered by Wnt/β-catenin signaling are inherently tolerant to the cytotoxic insult, allowing them to survive initial therapy and seed subsequent disease recurrence [108,109,110,111,112].

#### 3.4.2. Tumor Microenvironment Crosstalk and ADC Resistance

Wnt/β-catenin pathway promotes stemness and antigen heterogeneity, indirectly reducing ADC targeting efficiency and conferring resistance to payload cytotoxicity. Beyond promoting cellular stemness, the Wnt/β-catenin pathway actively interacts with the TME to establish specialized resistance mechanisms against ADCs. In hepatocellular carcinoma (HCC), the Wnt network regulates the interplay between tumor cells and local immune populations, crafting an immune-tolerant microenvironment that blunts the secondary immunogenic cell death often triggered by ADC payloads [66,67].

**Figure 5:**
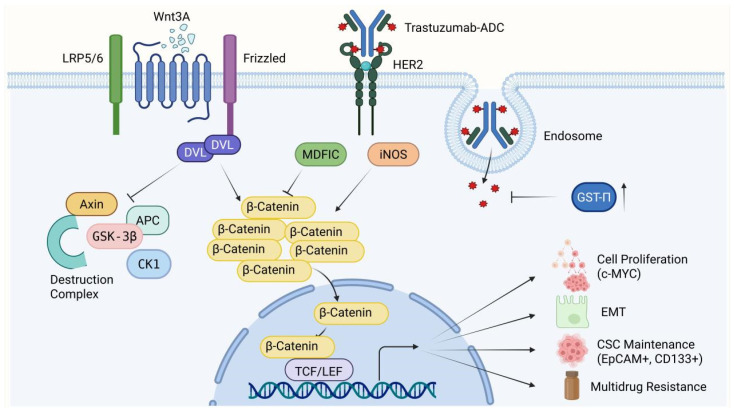
The Wnt/β-catenin signaling axis and the mechanism of action of Trastuzumab-ADC.

Furthermore, the pathway engages in direct biochemical neutralization of therapeutic agents. In NSCLC, the activation of the Wnt/β-catenin pathway by inducible nitric oxide synthase (iNOS) leads to the direct upregulation of enzymatic detoxification proteins, such as glutathione S-transferase pi GST−π and topoisomerase II alpha (TOPO IIα) [68]. This presents a unique and formidable resistance mechanism: even when an ADC successfully internalizes and releases its payload, Wnt-driven detoxification enzymes chemically neutralize the payload before it can bind to its intracellular targets. These findings highlight that evaluating ADC efficacy must account for the intracellular enzymatic landscape dictated by Wnt signaling (Figure 5).

The Wnt signaling pathway is primarily activated by the binding of the Wnt3A ligand to the Frizzled receptor and the LRP5/6 co-receptors. This interaction activates Dishevelled (DVL), which in turn inhibits the destruction complex composed of Axin, APC, GSK-3β, and CK1, thereby preventing the degradation of β-catenin. The cytoplasmic accumulation of β-catenin is negatively regulated by MDFIC and positively regulated by iNOS. The stabilized β-catenin subsequently translocates to the nucleus and interacts with TCF/LEF transcription factors to drive downstream effects, including cell proliferation (via c-MYC), EMT, CSC maintenance, and multidrug resistance. However, in the context of targeted therapy, Trastuzumab-ADC binds to HER2 and undergoes internalization and lysosomal/endosomal degradation, releasing a cytotoxic payload intended to induce cell death. Nevertheless, resistance can occur through the upregulation of GST-π, which inhibits the efficacy of the released payload. Collectively, the modulation of the Wnt signaling pathway and enzymatic detoxification processes jointly determine the balance between tumor cell survival and therapy-induced apoptosis. Abbreviations: HER2: human epidermal growth factor receptor 2; ADC: antibody–drug conjugate; LRP5/6: low-density lipoprotein receptor-related protein 5/6; DVL: Dishevelled; APC: adenomatous polyposis coli; GSK-3β: glycogen synthase kinase-3 beta; CK1: casein kinase 1; MDFIC: MyoD family inhibitor domain-containing protein; iNOS: inducible nitric oxide synthase; TCF/LEF: T-cell factor/lymphoid enhancer factor; GST-π: glutathione S-transferase pi; EMT: epithelial–mesenchymal transition; CSC: cancer stem cell; EpCAM: epithelial cell adhesion molecule.

#### 3.4.3. Clinical Translation Challenges and Future Therapeutic Strategies

The pervasive crosstalk between the Wnt/β-catenin pathway and other oncogenic networks makes it a challenging target for clinical translation. Because Wnt signaling drives both the maintenance of cancer stemness and direct payload detoxification, resolving this resistance requires precise intervention. However, the ubiquitous physiological role of Wnt signaling necessitates highly selective, next-generation pathway inhibitors to avoid severe off-target toxicities [113]. Future clinical strategies must prioritize mapping patient-specific Wnt/TME interactions to safely sequence Wnt inhibitors alongside ADCs, aiming to eradicate the stem-like, drug-tolerant persister cells that dictate clinical relapse (Table 1).

### 3.5. The RTK Signaling Pathway

#### 3.5.1. Structural Function and Oncogenic Activation Patterns of Receptor Tyrosine Kinases

Receptor tyrosine kinases (RTKs)—including the EGFR, HER, and MET families—are structurally composed of an extracellular ligand-binding domain, a transmembrane region, and an intracellular kinase domain. Because ADCs physically rely on extracellular domains for precise docking, RTK structural integrity and expression patterns are critical. Tumors frequently acquire mutations in the extracellular domain that alter antibody binding epitopes or impede the receptor’s endocytic internalization, directly neutralizing the ADC’s primary mechanism of action [114,115,116].

Furthermore, oncogenic mutations within the transmembrane or kinase domains can drive ligand-independent, constitutive activation of parallel survival networks, protecting the cell from the apoptosis intended by the internalized payload [117]. Recently, it has been discovered that RTK fusion proteins can form dense cytoplasmic biomolecular condensates. These condensates physically decouple oncogenic signaling from the plasma membrane, fundamentally altering antigen localization and shielding the signaling machinery from surface-targeting ADCs [118].

#### 3.5.2. Mechanisms of ADC Resistance Mediated by RTK Reprogramming and Bypass Signaling Activation

The sheer redundancy of the RTK network acts as a primary fail-safe for tumor cells, leading to a resistance phenomenon known as RTK reprogramming. When an ADC successfully suppresses its primary RTK target, the tumor dynamically shifts its dependency to alternative surface receptors to maintain signaling flux [69].

**Figure 6:**
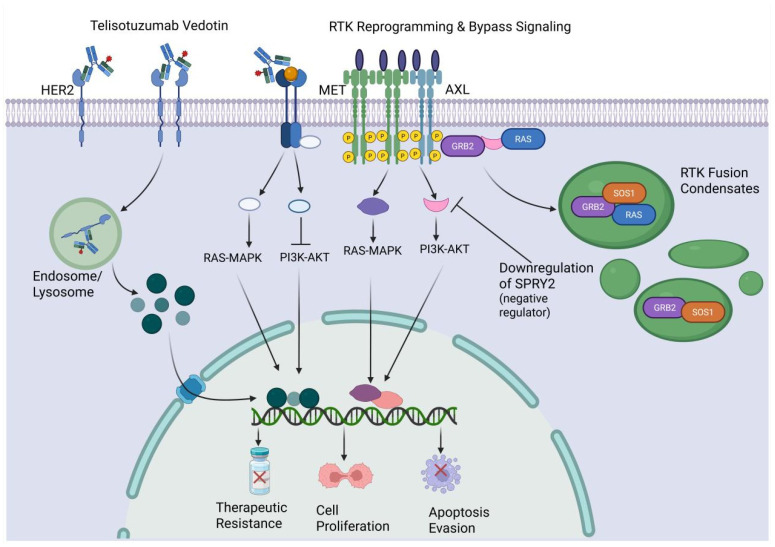
Telisotuzumab Vedotin mechanism of action and resistance via RTK reprogramming.

This bypass signaling is observed across a multitude of malignancies. For example, in EGFR-mutant NSCLC, prolonged therapeutic pressure frequently selects for MET gene amplification, effectively bypassing the EGFR blockade. Recognizing this adaptive shift, researchers are now deploying MET-targeting ADCs to exploit this exact resistance mechanism, turning the tumor’s new bypass receptor into a fresh therapeutic vulnerability [70]. A similar redundancy is observed in anaplastic lymphoma kinase (ALK)-rearranged lung cancers, where resistance is mediated by a compensatory surge in EGFR, MET, and insulin-like growth factor 1 receptor (IGF1R) [71]. In HER2-positive breast cancer, the inhibition of HER2 can trigger an autocrine loop that drives compensatory HER3-EGFR-PI3K signaling, underscoring why single-receptor targeting is rarely curative [72,73]. Analytically, these data demonstrate that tumor cells survive not merely by mutating the primary target, but by rerouting the signaling channel. (Figure 6).

The therapeutic process is primarily initiated by the binding of the ADC Telisotuzumab Vedotin to HER2, which undergoes internalization into endosomes and lysosomal degradation. This process releases a cytotoxic payload that targets the nucleus to induce therapeutic effects. However, resistance can occur through mechanisms such as RTK reprogramming and bypass signaling involving the activation of receptors like MET and AXL. These receptors recruit adaptor proteins such as GRB2 to stimulate downstream cascades, including the RAS-MAPK and PI3K-AKT pathways. This signaling network is further amplified by the downregulation of the negative regulator SPRY2 and the formation of RTK fusion condensates. These condensates, containing SOS1, GRB2, and RAS, concentrate signaling components to enhance pathway activation. Through these combined mechanisms, the modulation of bypass signaling and condensate formation drives cell proliferation and apoptosis evasion, ultimately resulting in therapeutic resistance. HER2: human epidermal growth factor receptor 2; RTK: receptor tyrosine kinase; MET: mesenchymal–epithelial transition factor; AXL: AXL receptor tyrosine kinase; GRB2: growth factor receptor-bound protein 2; RAS: rat sarcoma virus; MAPK: mitogen-activated protein kinase; PI3K: phosphoinositide 3-kinase; AKT: protein kinase B; SPRY2: sprouty homolog 2; SOS1: son of sevenless homolog 1.

#### 3.5.3. Combination Therapy Strategies and Precision Medicine Prospects for Overcoming Resistance Mediated by RTK Signaling Pathways

Because RTK-mediated resistance relies on the activation of bypass signaling and compensatory feedback loops, the clinical paradigm must shift from reactive sequential monotherapy to proactive combination strategies. Overcoming this resistance requires the simultaneous blockade of both the primary oncogenic driver and the anticipated emergent bypass routes.

To achieve this, clinical trials are increasingly evaluating dual-modality approaches, such as combining surface-targeting ADCs with broad-spectrum tyrosine kinase inhibitors (TKIs). By actively suppressing the entire compensatory RTK signaling network, TKIs prevent the establishment of feedback loops, thereby locking the tumor in a vulnerable state and maximizing the cytotoxic impact of the ADC [119]. Furthermore, the development of bispecific ADCs—capable of simultaneously binding multiple RTKs (e.g., EGFR and HER3)—represents the next evolutionary step in precision oncology, offering a mechanism to physically trap and degrade multiple resistance nodes simultaneously [70,71,120] (Table 1).

## 4. Emerging Signaling Nodes and Druggable Targets

### 4.1. Discovery and Validation of Novel ADC Targets

The integrated preclinical platform developed by Champions Oncology represents an advanced translational research infrastructure for ADC development. Specifically, this platform comprises a highly characterized, large-scale repository of patient-derived xenograft (PDX) models integrated with comprehensive multi-omics datasets, including genomics, transcriptomics, and proteomics. By leveraging these clinically relevant tumor models in conjunction with computational analytics and functional assays, researchers can systematically evaluate the in vivo efficacy of novel ADCs, identify predictive biomarkers for patient stratification, and elucidate molecular mechanisms underlying acquired resistance [121,122]. In addition, configurable multiplex immunofluorescence (mIF) platforms further enhance target validation and biomarker assessment. Through technologies such as immuno-spatial phenotyping (ISP) and artificial intelligence (AI)-driven image analysis, these systems enable high-resolution spatial profiling of tumor microenvironments with improved specificity, sensitivity, and reproducibility, thereby strengthening the translational applicability of ADC optimization strategies [123].

Despite significant advances in ADC development, discrepancies between preclinical efficacy and clinical outcomes remain a major translational challenge. One key contributing factor is the reliance on oversimplified tumor models. Conventional two-dimensional (2D) cell cultures fail to recapitulate tumor heterogeneity, spatial architecture, and dynamic interactions with the tumor microenvironment (TME). Similarly, traditional xenograft models often lack functional immune components and do not fully reflect human tumor biology, leading to an overestimation of therapeutic efficacy [22,121].

In contrast, more advanced preclinical platforms, such as patient-derived xenografts (PDX), organoids, and multi-omics-integrated models, offer improved physiological relevance by preserving tumor heterogeneity, stromal interactions, and molecular complexity. These models enable more accurate evaluation of ADC pharmacodynamics, payload delivery efficiency, and resistance mechanisms [121,122]. However, even these systems have limitations, including high cost, variability, and limited scalability.

Therefore, integrating advanced modeling systems with computational approaches and real-time biomarker analysis may provide a more reliable framework for predicting clinical responses. Future ADC development will benefit from such translationally relevant platforms to bridge the gap between preclinical success and clinical efficacy.

### 4.2. Novel Druggable Target

To expand the clinical utility of ADCs and overcome existing resistance mechanisms, novel targets and engineering strategies are being extensively explored across various solid tumors. For instance, carcinoembryonic antigen-related cell adhesion molecule 5 (CEACAM5) is frequently overexpressed in gastrointestinal and respiratory malignancies, making it a highly attractive target. SGN-CEACAM5C (SAR445953), a novel topoisomerase I inhibitor-based ADC, leverages this overexpression to deliver potent, selective, and dose-dependent antitumor activity in preclinical models of colorectal, pancreatic, gastric, and lung cancers [124]. Its robust bystander effect facilitates tumor regression across multiple PDX cohorts, achieving favorable disease control [124]. Similarly, M9140 is another CEACAM5-targeted ADC designed to address the specific challenge of heterogeneous target expression commonly observed in colorectal cancer [125]. Crucially, unlike traditional ADC payloads, M9140’s efficacy is not compromised by MDR-1-mediated drug efflux, offering a distinct advantage in resistant tumor settings [125].

Beyond single-antigen targeting, bispecific ADCs are being developed to overcome resistance driven by signaling crosstalk in epithelial tumors. PM1300 is a novel asymmetric immunoglobulin G (IgG)-like bispecific ADC targeting both EGFR and HER3. By exhibiting optimized affinity and selective binding specifically to EGFR/HER3 double-positive cancer cells, it minimizes off-target toxicity against EGFR single-positive cells. This dual-targeting approach yields potent antitumor efficacy and an improved safety profile in preclinical models [126]. Another promising pan-tumor strategy involves targeting B7-H3, an immune checkpoint molecule broadly upregulated in various solid tumors. YL201 and DB-1311 are novel ADCs targeting B7-H3. YL201 utilizes the innovative TMALIN platform to achieve high stability, efficient payload delivery, and broad-spectrum antitumor efficacy [127]. Meanwhile, DB-1311 employs a novel DNA topoisomerase I inhibitor with an optimized drug-to-antibody ratio (DAR) of 6, demonstrating enhanced cytotoxic potency and improved pharmacokinetics compared to existing analogues [128].

In the context of gastrointestinal cancers, Claudin 18.2 (CLDN18.2) has emerged as a highly specific target, with robust expression observed in 30–40% of gastroesophageal adenocarcinomas [129]. Clinical progress in this indication has been significant; updated Phase III trial data in 2024 demonstrated that a Zolbetuximab-monomethyl auristatin E (MMAE) conjugate achieved an 18% complete response rate in CLDN18.2-positive patients, significantly outperforming the paclitaxel control arm [130]. However, clinical efficacy in these gastric tumors is often limited by a unique resistance mechanism: isoform switching. When tumors co-express both CLDN18.1 and CLDN18.2, the formation of heteromeric tight junctions leads to a 67% reduction in ADC binding affinity [131,132]. To counter this resistance mechanism, genetic screening has identified that inhibition of PAK4 can restore CLDN18.2 membrane localization, thereby re-sensitizing gastric tumors to ADC therapy [133,134] (Table 2).

## 5. Clinical Translation Strategies and Ongoing Trials

### 5.1. Overview of ADC Drug Development and Technology Platform Evolution

The landscape of targeted oncology has been profoundly transformed by ADCs. As of 2025, fifteen ADC therapeutics have received FDA approval, collectively covering more than fifteen distinct cancer subtypes [135]. The momentum in this field is underscored by the fact that more than one hundred ADCs are currently under clinical development, with forty-one of them already advancing to Phase III trials [136]. This rapid clinical expansion is driven by the swift iteration of ADC technology platforms, which have progressed from early first-generation constructs to highly sophisticated third- and fourth-generation technologies. Notably, the DXd (deruxtecan) technology platform has emerged as one of the most successful frameworks, providing a robust foundation for numerous DXd-based ADCs currently advancing through clinical development across various solid tumors [137].

### 5.2. Biomarker-Guided Precision Therapy and Drug Resistance Monitoring

As ADCs are deployed across a wider range of malignancies, biomarker-guided precision treatment strategies have become central to personalizing therapy and anticipating resistance. In terms of predictive biomarker development, quantifying the expression levels of surface antigens such as HER3, Trophoblast cell-surface antigen 2 (TROP2), and HER2 has been validated to predict efficacy in breast, lung, and gastrointestinal cancers. Furthermore, in specific clinical scenarios like NSCLC, stratification strategies based on EGFR-dependent and EGFR-independent resistance mechanisms are providing crucial insights for clinical decision-making [135,138,139]. To track these dynamic resistance profiles, liquid biopsy technology is increasingly utilized for real-time monitoring of tumor evolution, facilitating timely adjustments to treatment regimens [140,141,142,143,144]. Despite these advances, a significant unmet need remains for highly specific biomarkers that can reliably predict both ADC efficacy and off-target toxicity, requiring deeper mechanistic investigation and larger-scale clinical validation.

### 5.3. Combination Therapy Strategies and Innovations in Clinical Trial Design

To further maximize efficacy and overcome acquired resistance in refractory tumors, combination therapy represents another critical direction in ADC development. Because ADCs can induce immunogenic cell death and release neoantigens, combining them with immune checkpoint inhibitors (ICIs) generates potent synergistic effects. Both preclinical and clinical studies have underscored the substantial potential of this combinatorial approach across diverse immunologically “cold” tumors [145,146,147]. Concurrently, to bypass specific bypass-track resistance mechanisms, combining ADCs targeting EGFR or HER2 with corresponding TKIs has proven effective, particularly in EGFR-mutated NSCLC or HER2-positive breast cancer. Current research focuses heavily on optimizing the dosing sequence and combination ratios of these regimens [148,149,150]. To efficiently evaluate these complex combination strategies across multiple tumor types, adaptive trial methodologies are being integrated into clinical study designs. By dynamically modifying trial protocols based on interim analyses and integrating multiple endpoints (such as ORR, PFS, overall survival(OS), and safety), these designs enhance trial success rates [151,152,153]. Moreover, basket and umbrella trials now enable patient selection based on molecular biomarkers rather than the tumor’s site of origin, providing a novel and highly efficient framework for cross-cancer type evaluation [135,154].

An additional challenge in ADC development lies in optimizing dosing strategies in the context of pharmacokinetic (PK) and pharmacodynamic (PD) variability. ADCs exhibit complex PK/PD profiles due to their multi-component structure, including antibody distribution, linker stability, and payload release kinetics. Variability in antigen expression, tumor penetration, and systemic metabolism further contributes to inter-patient differences in therapeutic response and toxicity. For instance, premature payload release may increase off-target toxicity, whereas insufficient intracellular drug accumulation may compromise efficacy. Moreover, the narrow therapeutic window of many ADCs necessitates careful dose optimization to balance efficacy and safety. Emerging strategies, including biomarker-guided dosing, adaptive treatment scheduling, and quantitative PK/PD modeling, are being explored to improve clinical outcomes and personalize ADC therapy [1,5,135]

### 5.4. Key Clinical Research Advancements and Next-Generation ADC Technologies

Addressing the pressing clinical challenge of targeted therapy resistance, recent ADC advancements have focused heavily on specific refractory solid tumors. A prime example is the treatment of patients with EGFR-mutated advanced NSCLC who have progressed on EGFR-TKIs. For this specific population, patritumab deruxtecan (HER3-DXd) has emerged as a highly promising HER3-targeting ADC designed to bypass traditional kinase resistance pathways. The HERTHENA-Lung01 trial, a global multicenter phase II study evaluating HER3-DXd [136], demonstrated a confirmed ORR of 32.4% (11/34) in NSCLC patients with EGFR-dependent resistance mechanisms, and 27.2% (22/81) in those with solely EGFR-independent mechanisms [140]. Subsequently, the HERTHENA-Lung02 trial achieved a key milestone in September 2024, demonstrating a statistically significant improvement in progression-free survival (PFS) for HER3-DXd compared to doublet chemotherapy in this patient cohort [142]. The median PFS reached 5.8 months, with significant improvements observed at the 6-, 9-, and 12-month timepoints [5].

Beyond NSCLC, ADCs are demonstrating broad therapeutic potential across other epithelial malignancies. In January 2025, the TROP2-targeting ADC datopotamab deruxtecan received FDA approval for a new indication, marking another significant milestone for targeting widely expressed pan-tumor antigens [155]. Simultaneously, to tackle the complex resistance networks driven by receptor crosstalk in heterogeneous tumors, bispecific ADCs are advancing rapidly. PM1300, a novel asymmetric IgG-like bispecific ADC targeting both EGFR and HER3, specifically addresses this by exhibiting optimized affinity and selective binding to EGFR/HER3 double-positive cancer cells, while sparing EGFR single-positive normal cells in preclinical models [142]. Ultimately, underpinning all these clinical and preclinical milestones are precise linker design strategies. The continuous optimization of linker stability and cleavability remains crucial, as next-generation linker technologies are fundamentally responsible for improving the pharmacokinetic profiles and widening the therapeutic index of modern ADCs.

In contrast to conventional ADC strategies, logic-gated delivery systems are emerging as a promising approach to enhance the precision and safety of ADCs. These systems are designed to enable payload release only under specific combinatorial conditions, such as the presence of multiple tumor-associated antigens or distinct microenvironmental cues (e.g., pH, enzymatic activity). By requiring dual- or multi-input signals, logic-gated carriers can significantly reduce off-target toxicity while improving tumor selectivity. This strategy is particularly advantageous in heterogeneous tumors, where single-antigen targeting is often insufficient to achieve durable responses. Integrating logic-gated design principles with next-generation ADC platforms may further refine targeted delivery and enhance the therapeutic index [153,156].

## 6. Future Perspectives

Looking ahead, the therapeutic landscape of ADCs is evolving from simple cytotoxic delivery toward sophisticated, multi-functional precision tools. To address the persistent challenges of tumor heterogeneity and acquired resistance, next-generation ADCs with dual-targeting properties are emerging. A prominent example is ZW49 (targeting both HER2 and EGFR), which is designed to prevent tumor escape in epithelial cancers where single-antigen loss or compensatory signaling frequently occurs [156,157,158]. In parallel, the scope of ADC therapy is broadening to include previously “undruggable” targets through innovative payloads, such as non-cytotoxic proteolysis-targeting chimeras (PROTACs) conjugated to antibodies [159,160]. Furthermore, to move beyond the limitations of conventional immunohistochemistry, diagnostic advances like high-resolution positron emission tomography (PET) imaging with radiolabeled antibodies (e.g., Zr-trastuzumab) are enabling superior patient stratification [161]. These technological leaps support a global ADC market projected to exceed $16.5 billion by 2030, fueled by the move into earlier-line settings and the targeting of refractory-specific antigens such as B7-H3 and delta-like canonical Notch ligand 3 (DLL3) [162,163,164,165].

Mechanistically, overcoming ADC resistance requires a multi-pronged approach that addresses the multifactorial nature of treatment failure, including antigen loss, payload efflux, and intracellular trafficking defects [166]. As discussed throughout this review, the hyperactivation of PI3K/AKT and MAPK pathways remains a central pillar of resistance by promoting cell survival and upregulating drug efflux pumps like P-gp [23]. To counteract this, third-generation ADC platforms are being engineered with improved linker stability and novel payloads capable of bypassing MRP-mediated resistance. Importantly, the future of ADC therapy lies in rational combination strategies tailored to the tumor’s molecular landscape. This includes pairing ADCs with PI3K/AKT/mTOR or MAPK inhibitors to block escape pathways, or utilizing mTOR inhibitors to specifically enhance lysosomal processing of the ADC [23,44]. Additionally, combining ADCs with immune checkpoint blockade can leverage the release of neoantigens to augment systemic anti-tumor immunity (110). These adaptive strategies will be increasingly guided by liquid biopsy technologies, allowing for the real-time tracking of resistance evolution.

Another important direction for next-generation ADC development involves integrating tumor stress response mechanisms into therapeutic design. ADC-induced cytotoxic stress can activate adaptive survival pathways, including autophagy, ferroptosis resistance, and immune exhaustion, which collectively enable tumor persistence under treatment pressure. For example, autophagy may facilitate the recycling of damaged cellular components, while ferroptosis resistance mechanisms prevent lipid peroxidation-induced cell death [167]. In parallel, prolonged antigen exposure and inflammatory signaling may contribute to T-cell exhaustion within the tumor microenvironment. Targeting these adaptive responses—either through combinatorial therapies or through rational ADC engineering—may enhance treatment efficacy. Future ADC strategies may therefore incorporate stress-modulating agents or immunoregulatory components to overcome these resistance mechanisms and achieve more durable responses.

The transition toward next-generation targets is already yielding significant clinical results in traditionally difficult-to-treat malignancies. For instance, in metastatic settings where single-agent efficacy is often limited, the combination of the Nectin-4 ADC 9MW2821 plus toripalimab achieved a remarkable ORR of 87.5% [168]. Similarly, in small-cell lung cancer (SCLC)—a disease characterized by rapid progression and high recurrence—BL-B01D1 has shown encouraging activity with an ORR of 55.2% [169]. The expansion into pancreatic ductal adenocarcinoma (PDAC), one of the most stroma-rich and resistant solid tumors, is also gaining momentum with CLDN18.2-targeting agents like IBI343 [170]. Furthermore, for advanced solid tumors driven by MET alterations, the MET-directed ADC SHR-1826 has achieved an ORR of 39.7% [171]. Collectively, these advancements underscore a paradigm shift in oncology toward multi-target engineering, biomarker-guided precision combinations, and dynamic resistance monitoring, promising a more durable therapeutic index for cancer patients [23,166,172,173] (Table 3).

## 7. Conclusions

As oncology increasingly advances toward precision medicine, ADCs are emerging as a cornerstone of targeted cancer therapy. However, the durability of these treatments is frequently compromised by adaptive oncogenic signaling rewiring. This review has delineated how major intracellular cascades—specifically the PI3K/AKT/mTOR, MAPK/ERK, Wnt/β-catenin, RTK, and JAK/STAT3 pathways—serve as the molecular engine for ADC resistance. Mechanistically, hyperactivation of the PI3K/AKT/mTOR and MAPK/ERK axes promotes survival by upregulating drug efflux pumps (e.g., P-gp) driving EMT, and remodeling the tumor microenvironment. These findings provide a strong clinical rationale for incorporating specific pathway inhibitors into ADC-based regimens to resensitize resistant cells [23,166].

In parallel, we highlight the role of complex crosstalk in maintaining tumor viability under ADC-induced stress. For instance, ROR1-mediated Wnt5a signaling creates a robust resistance network by simultaneously activating the PI3K/AKT, MAPK, NF-κB, STAT3, and Hippo pathways [166]. This is particularly evident in NSCLC, where persistent hyperactivation of canonical Wnt/β-catenin signaling sustains cancer stemness and multidrug resistance, explaining why traditional single-target ADCs often fail in this indication [14,174]. Furthermore, the inherent redundancy within RTK signaling—especially aberrant EGFR/HER family activation—enables tumor cells to bypass ADC-mediated cytotoxicity via alternative growth signals [74]. Similarly, in bladder carcinoma, where ADCs have recently revolutionized care, dysregulation of the JAK/STAT3 pathway further supports tumor survival and immune evasion, marking it as a critical actionable node for biomarker-guided combination strategies [18,19].

To translate these mechanistic insights into clinical success, next-generation ADC platforms are being engineered to preemptively tackle these resistance nodes. Among these advances, multi-specific ADCs represent a key strategy for overcoming tumor heterogeneity and adaptive resistance. Unlike conventional monospecific ADCs, multi-specific constructs can simultaneously target multiple antigens or signaling pathways, thereby reducing the likelihood of antigen escape and compensatory signaling activation. This approach is particularly relevant in tumors characterized by heterogeneous antigen expression or dynamic receptor reprogramming. By engaging multiple targets, multi-specific ADCs can enhance binding avidity, improve internalization efficiency, and disrupt redundant survival networks. Recent developments in bispecific and trispecific antibody engineering further support the feasibility of this strategy.

In addition to multi-targeting approaches, innovations include dual-payload ADCs designed to bypass efflux-mediated resistance and immunostimulatory antibody conjugates (ISACs) to reprogram the immunosuppressive microenvironment [23,173,175]. The clinical viability of these “pathway-informed” strategies is already being realized; for example, in HER2-positive breast cancer—a disease where PI3K mutations often lead to T-DM1 failure—the combination of T-DM1 plus the PI3K inhibitor alpelisib achieved a promising 43% objective response rate [176]. Ultimately, the future of ADC therapy will depend on the synergy between advanced engineering—such as site-specific conjugation and linker/payload innovation—and the implementation of precision, molecularly tailored treatment regimens to overcome the evolving landscape of resistant cancers [23,176].

Collectively, these advances underscore a paradigm shift from empirically designed ADCs toward mechanism-driven, pathway-informed therapeutic strategies, paving the way for more durable and personalized anticancer treatments.

## Figures and Tables

**Table 1:** Signaling Pathways and Secondary Resistance Mechanisms to ADCs.

Signaling Pathway	Secondary Resistance Mechanism	Specific Molecular Alteration/Process	Impact on ADC Efficacy	References
PI3K/AKT/mTOR	Apoptosis Inhibition & Drug Efflux	Constitutive Activation: Mutations (e.g., PIK3CA) or PTEN loss drive survival signals that counteract payload-induced cytotoxicity. Efflux Pump Upregulation: Activation of AKT enhances P-gp expression.	Prevents cytotoxic payload from inducing cell death; reduces intracellular drug concentration (e.g., T-DM1 resistance).	[37,38,39,40,41,42,43,45,46,47,52,53]
MAPK/ERK	Drug efflux & Survival compensation	Trafficking Impairment: Altered recycling or internalization of surface antigens (e.g., Trop-2). Feedback Activation: Inhibition of primary targets leads to compensatory RAS-RAF-MEK activation.	Reduces lysosomal delivery of the ADC; maintains proliferation despite target blockade (e.g., Sacituzumab govitecan resistance).	[54,55,56,57,58]
JAK/STAT3	Antigen Downregulation & TME Remodeling	Antigen Loss: IL-6/STAT3 signaling downregulates surface targets like CD38. Efflux Transporters: Upregulates ABC transporters (P-gp, ABCG2). Immune Evasion: Recruits MDSCs/M2 macrophages.	Decreases ADC binding sites (e.g., Anti-CD38 ADCs); actively expels payload; suppresses bystander immune effects.	[18,19,59,60,61,62,63,64,65]
Wnt/β-catenin	Stemness Maintenance & Payload Detoxification	CSC Enrichment: Maintains EpCAM+ CSCs via β-catenin stabilization. Enzymatic Detoxification: iNOS-driven Wnt activation regulates GST-π.	Promotes a drug-tolerant stem-like state; GST-π neutralizes the cytotoxic payload.	[14,15,66,67,68]
RTK Signaling (EGFR/HER/MET)	Bypass Signaling & Heterogeneity	Alternative Pathway Activation: Activation of MET, AXL, or HER3 bypasses the blockade of the primary ADC target (e.g., EGFR or HER2). Co-expression: Heterogeneous expression of RTKs allows survival via non-targeted receptors.	Tumor cells survive via secondary RTK networks despite effective engagement of the primary ADC target (e.g., HER3-mediated resistance to T-DM1).	[16,17,69,70,71,72,73,74]

**Abbreviations**: **ABC**, ATP-binding cassette; **ABCG2**, ATP-binding cassette super-family G member 2; **ADC**, antibody–drug conjugate; **AKT**, protein kinase B; **AXL**, AXL receptor tyrosine kinase; **CD38**, cluster of differentiation 38; **CSC**, cancer stem cell; **EGFR**, epidermal growth factor receptor; **EpCAM**, epithelial cell adhesion molecule; **ERK**, extracellular signal-regulated kinase; **GST-π**, glutathione S-transferase pi; **HER**, human epidermal growth factor receptor; **HER3**, human epidermal growth factor receptor 3; **IL-6**, interleukin-6; **iNOS**, inducible nitric oxide synthase; **JAK**, Janus kinase; **MAPK**, mitogen-activated protein kinase; **MDSC**, myeloid-derived suppressor cell; **MEK**, mitogen-activated protein kinase kinase; **MET**, mesenchymal–epithelial transition factor; **mTOR**, mammalian target of rapamycin; **P-gp**, P-glycoprotein; **PI3K**, phosphoinositide 3-kinase; **PIK3CA**, phosphatidylinositol-4,5-bisphosphate 3-kinase catalytic subunit alpha; **PTEN**, phosphatase and tensin homolog; **RAS-RAF**, rat sarcoma virus-rapidly accelerated fibrosarcoma; **RTK**, receptor tyrosine kinase; **STAT3**, signal transducer and activator of transcription 3; T-DM1, trastuzumab emtansine; **TME**, tumor microenvironment; **Trop-2**, trophoblast cell-surface antigen 2.

**Table 2:** Selected examples of approved ADC therapies targeting cancer.

ADC Drug	Target	Tumor Type	Main Indication	Resistance Pathways
anti-CD38	CD38	Multiple Myeloma	Relapsed/Refractory Multiple Myeloma	CD38 downregulation by the JAK/STAT3 pathway as a mechanism of immune evasion and drug resistance [59,60,61,62,63,64,65]
Trastuzumab emtansine (T-DM1)	HER2	HER2-positive Breast Cancer	Breast Cancer	PI3K/AKT/mTOR pathway activation [45,46,47,52,53]
Sacituzumab govitecan	Trop-2	Various solid tumors (e.g., Triple Negative Breast Cancer)	Metastatic breast cancer	MAPK/ERK signaling, EMT, stem-like properties [54,55,56,57]
T-DXd (Trastuzumab Deruxtecan)	HER2	HER2-positive Breast Cancer, Gastric Cancer	Breast and gastric cancers	Wnt/β-catenin pathway, PI3K/AKT/mTOR pathway [67,68,113,114,115,116,117,118,125,126]
Enfortumab vedotin (EV)	Nectin-4	Urothelial carcinoma, metastatic solid tumors	Urothelial carcinoma	RTK bypass signaling [69,125,126,135]

**Abbreviations**: **ADC**, antibody–drug conjugate; **AKT**, protein kinase B; **CD38**, cluster of differentiation 38; **EMT**, epithelial–mesenchymal transition; **ERK**, extracellular signal-regulated kinase; **EV**, enfortumab vedotin; **HER2**, human epidermal growth factor receptor 2; **JAK**, Janus kinase; **MAPK**, mitogen-activated protein kinase; **mTOR**, mammalian target of rapamycin; **PI3K**, phosphoinositide 3-kinase; **RTK**, receptor tyrosine kinase; **STAT3**, signal transducer and activator of transcription 3; **T-DM1**, trastuzumab emtansine; **T-DXd**, trastuzumab deruxtecan; **Trop-2**, trophoblast cell-surface antigen 2.

**Table 3:** Future Perspectives on ADC Development, Resistance Management, and Clinical Advances.

Domain	Key Developments & Mechanisms	Clinical Implications & Future Outlook	References
Technological Innovation	Next-Gen Designs: Dual-targeting ADCs (e.g., ZW49 targeting HER2/EGFR). Novel Payloads: Non-cytotoxic payloads (e.g., PROTAC-ADCs). 3rd-Gen Tech: Enhanced linker stability, precision targeting, and potent payloads to bypass MRP-mediated resistance.	Addresses tumor heterogeneity, therapeutic resistance, and targets previously considered “undruggable.” Improves therapeutic index.	[23,156,157,158,159,160]
Diagnostics & Market	Imaging: Shift from IHC to high-resolution PET using radiolabeled antibodies (e.g., 89Zr-trastuzumab). Market Growth: Forecast to exceed $16.5 billion by 2030 (CAGR > 10%).	Enables precise patient selection. Market expansion driven by neoadjuvant settings and novel targets (B7-H3, DLL3) in refractory cancers.	[161,162,163,164,165].
Resistance Mechanisms	Pathways: Activation of PI3K/AKT and MAPK axes promotes survival and P-gp upregulation (multidrug resistance). Factors: Antigen loss, payload resistance, antibody alterations, and trafficking defects.	Highlights the need to move from empirical application to multi-dimensional strategies focusing on core signaling networks.	[23,166]
Therapeutic Strategies	Combinations: ADCs + PI3K/AKT/mTOR inhibitors (promote lysosomal processing) or Immune Checkpoint Inhibitors (enhance immunogenicity). Multi-targeting: Bispecific ADCs to counter target loss. Monitoring: Liquid biopsy for real-time tracking of resistance evolution.	Shift from static treatment to dynamic, biomarker-guided precision medicine. Synergistic effects observed in overcoming resistance.	[23,44]
Emerging Clinical Data	9MW2821 (Nectin-4) + Toripalimab: UC (1L), ORR 87.5%, DCR 92.5%. Effective in low-expression/liver mets. BL-B01D1: SCLC, ORR 55.2%, Median overall survival (mOS) 12.0 months. IBI343 (CLDN18.2): PDAC, ORR 22.7%, DCR 81.8% (CLDN18.2+). SHR-1826 (MET): Advanced solid tumors, ORR 39.7%, DCR 94.8%.	Demonstrates significant efficacy across diverse and difficult-to-treat indications (UC, SCLC, PDAC), validating the potential of novel targets and combination regimens.	[168,169,170,171,172,173]

**Abbreviations**: **1L**, first-line; **ADC**, antibody–drug conjugate; **AKT**, protein kinase B; **B7-H3**, B7 homolog 3; **CAGR**, compound annual growth rate; **CLDN18.2**, claudin 18.2; **DCR**, disease control rate; **DLL3**, delta-like canonical Notch ligand 3; **EGFR**, epidermal growth factor receptor; **HER2**, human epidermal growth factor receptor 2; **IHC**, immunohistochemistry; **MAPK**, mitogen-activated protein kinase; **MET**, mesenchymal–epithelial transition factor; **mOS**, median overall survival; **MRP**, multidrug resistance-associated protein; **mTOR**, mammalian target of rapamycin; **ORR**, objective response rate; **PDAC**, pancreatic ductal adenocarcinoma; **PET**, positron emission tomography; **P-gp**, P-glycoprotein; **PI3K**, phosphoinositide 3-kinase; **PROTAC**, proteolysis targeting chimera; **SCLC**, small cell lung cancer; **UC**, urothelial carcinoma.

## Data Availability

No new data were created or analyzed in this study.

## Abbreviations

The following abbreviations are used in this manuscript:

ADC	Antibody–drug conjugate
RTK	Receptor tyrosine kinase
ERK	Extracellular signal-regulated kinase
FDA	Food and Drug Administration
HER2	Human epidermal growth factor receptor 2
PI3K	Phosphoinositide 3-kinase
MAPK	Mitogen-activated protein kinase
STAT3	Signal transducer and activator of transcription 3
T-DM1	Trastuzumab emtansine
EGFR	Epidermal growth factor receptor
NSCLC	Non-small cell lung cancer
TME	Tumor microenvironment
mAb	Monoclonal antibody
ABC	ATP-binding cassette
PTEN	Phosphatase and tensin homolog
ORR	Objective response rate
IC50	Half-maximal inhibitory concentration
IL-6	Interleukin-6
MDSC	Myeloid-derived suppressor cell
PD-L1	Programmed death-ligand 1
TKI	Tyrosine kinase inhibitor
PDX	Patient-derived xenograft
mIF	Multiplex immunofluorescence
TROP2	Trophoblast cell-surface antigen 2
PFS	Progression-free survival
mOS	Median overall survival
PK	pharmacokinetic
PD	pharmacodynamic
TNBC	Triple-negative breast cancer
